# Key Learning Points from a Case of Cannabinoid Induced Catatonia

**DOI:** 10.1192/bjo.2022.353

**Published:** 2022-06-20

**Authors:** Lydia Benazaize, Nieves Mercadillo

**Affiliations:** 1 Mersey Care NHS Foundation Trust, Liverpool, United Kingdom; 2 Warrington and Halton Hospitals NHS Foundation Trust, Warrington, United Kingdom

## Abstract

**Aims:**

Catatonia has an effective treatment: benzodiazepines. A first presentation of catatonia may present initially to an acute medical trust. It is important acute clinicians are familiar with its manifestations, medical differentials, and most importantly, understand the role of benzodiazepines in both the investigation and management of catatonia.

**Methods:**

Here we describe a case of catatonia in a nineteen-year-old male, who presented acutely to the accident and emergency department with odd behaviour following inhalation of the synthetic cannabinoid ‘spice’. Initially, he was found to be rigid, mute and doubly incontinent, but able to follow vague commands. He was admitted to the acute trust for twelve days in which he was worked-up as a case of drug induced psychosis. As he was not improving, he was then transferred to psychiatric inpatient services for further investigation and management.

**Results:**

The acute medical team did not recognise this as a presentation of catatonia and did not conduct a lorazepam challenge, as suggested by specialist services. A lorazepam challenge is helpful in both diagnosing and treating catatonia. In this case, we believe this may have been missed, due to a lack of knowledge and understanding of the condition. Medical mimics of psychosis, such as autoimmune encephalitis, may be life threatening, but have a good prognosis if treated early. Here, these were not considered, which may have led to disastrous consequences had they been present. This case shows an opportunity for education into the differentials and management of catatonia.

**Conclusion:**

We believe this case highlights a degree of poor understanding surrounding catatonia and its clinical work-up in the acute setting. There were missed opportunities to instigate treatment earlier and consider rarer alternative causes for the presentation. We hope this case will simplify diagnosis and management for acute clinicians, and highlight important medical mimics of catatonia. This case also shows the potential significant harms of synthetic cannabinoids such as ‘spice’ and highlights a need for further research and potential review for grading above Class B in the Misuse of Drugs act 1971.

